# Household exposure to pesticides and bladder exstrophy in a newborn baby boy: a case report and review of the literature

**DOI:** 10.1186/1752-1947-3-6626

**Published:** 2009-03-30

**Authors:** Marlene Martin, Kristina Rodriguez, Miguel Sánchez-Sauco, Gerardo Zambudio-Carmona, Juan Antonio Ortega-García

**Affiliations:** 1 Pediatric Environmental Health Specialty Unit, Hospital University Virgen de la Arrixaca, Carretera Madrid-Cartagena 30120 El Palmar, Murcia, Spain; 2 Pediatric Surgery and Urology, Hospital University Virgen de la Arrixaca, Carretera Madrid-Cartagena 30120 El Palmar, Murcia, Spain

## Abstract

**Introduction:**

Bladder exstrophy is a rare urogenital abnormality. Other urogenital malformations have been associated with exposure to hormonal pesticide disruptors during critical developmental periods. This is the first report in the literature to associate household exposure to pesticides with bladder exstrophy.

**Case presentation:**

We describe the pediatric environmental history of a newborn baby boy with isolated bladder exstrophy. In this case the pediatric environmental history includes the constitutional, genealogical, genetic and environmental factors related to bladder exstrophy, which revealed a cockroach infestation in the parents' home and the daily use of bug spray to kill them. The mother used one bottle of spray every 2 days (1000cc) and more in the summer, when the problem was worse. During gestational weeks 0-12, the mother intensively used a domestic pesticide consisting of a mixture of pyrethroids (cyfenothrin 0.5%, and tetramethrin 0.31%) and pyriproxyfen (0.01%). She described repeated episodes of mild to moderate poisoning that are associated with the use of household pesticides. The mother is a housewife and the father works as a fumigator of fruit fields and he reported gastrointestinal symptoms associated with the use of occupational pesticides. However, he did not believe he carried traces of these products into the home and his wife washed his work clothes separately. The pyrethroids and pyriproxyfen were detected in a urine sample obtained from the child 4 months after he was born. No other risk factors were identified.

**Conclusions:**

A detailed and carefully conducted pediatric environmental history, which includes information about home pesticide use, should be carried out for all children with bladder exstrophy. Domestic exposure to pesticides during critical developmental periods may have deleterious effects for the fetus.

## Introduction

Bladder exstrophy (BE) is a rare urogenital abnormality present in one out of every 30 000-50 000 live births. Although its etiology is unknown, several risk factors (RFs) have been identified or studied. The following RFs are associated with BE: summer conception, white, non-Hispanic maternal race/ethnicity; male sex; and intrauterine exposure to diazepam or diphenylhydantoin [1]-[3]. BE occurs more frequently in descendents of individuals who also have the disease and is associated with Opitz and Al Awadi-Raas-Rothschild syndromes [4]-[6]. Malformations of the urogenital tract are also related to exposure to hormonal disruptors. However, evidence that the suggested increase in male urogenital abnormalities in humans can be attributed to exposure to pesticides known to have hormone disrupting effects is limited [7]-[8].

The pediatric environmental history (PEH) forms part of the clinical record and is used to register the absence or presence of RFs associated with the occurrence of diseases such as BE. The PEH employs a series of basic and concise questions including genetic, genealogical and constitutional aspects that allow the clinicians to identify the environmental RFs [9]. The purpose of our case study is to illustrate the PEH´s role in identifying the RFs associated with BE and to explain the potential relationship between pesticide exposure and BE.

## Case presentation

We present the case of a newborn baby boy with BE focusing on the PEH (Table 1). He was born after 38-weeks gestation via a cesarean section and had an Apgar score of 10/10; the amniotic fluid was clear and his birth weight was 6lbs.

**Table 1:** The pediatric environmental history can be used to identify the absence or presence of risk factors associated with bladder exstrophy and other pediatric diseases

Constitutional & genealogic factors	• Sex
	• Race/ethnicity
	• Family history
	• Family tree
Reproductive history	• Pregnancies
	• Hormonal therapy
Environmental factors	• Socioeconomic status
	• Home
	• Community
	• Medical history of ionizing radiation
	• Legal (alcohol & tobacco) and illegal drug use
	• Pharmaceutics/medications
	• Occupational exposures
	• Hobbies
	• Home remedies/herbal supplements

The reproductive history, constitutional and genealogic factors, and environmental components form the basis of the pediatric environmental history and are shown here.

The findings of the neonatal physical examination included BE with a rudimentary gland, normal anus and rectum, undescended testicles present in the inguinal canal and a normal scrotum. No other abnormalities were found in chest, abdominal, cardiac or head examination. The patient's growth, phenotype and karyotype were otherwise normal. A family tree was constructed to first and second degree and showed no family history of malformations or rare diseases.

Both parents are of Moroccan origin and had lived on the first floor of a 25-year-old building in Santomera, Murcia, Spain since 2003. The mother is 32 years old with three previous pregnancies and no history of oral contraceptive use, and her menstrual periods had been regular. She has two daughters aged 6 and 1.5 years and had one abortion at 10-weeks' gestation during the summer of 2004. Her fourth pregnancy was spontaneous and unplanned (last menstrual period on 14 August 2006). This last pregnancy was normal until the 35^th^ week of gestation when a sonogram showed the possibility of BE.

The parents reported a cockroach infestation in their home and daily use of a bug spray to treat them. The mother used one bottle of spray every two days (1000cc). The problem was worse during the summer months, when her use of the spray increased. During gestational weeks 0-12, the mother intensively used the domestic pesticide consisting of a mixture of pyrethroids (cyfenothrin 0.5% and tetramethrin 0.31%) and pyriproxyfen (0.01%). The mother described repeated episodes of mild to moderate poisoning including mild headaches, general discomfort, rhinitis, sneezing, dyspnea, wheezing, dry and sore throat, conjunctivitis and a cough that coincides with the use of this pesticide.

The mother is a housewife and the father works as a fumigator in fruit fields and was unaware of precautions he could take to protect himself at work. He reported gastrointestinal symptoms associated with the use of occupational pesticides. However, he did not believe that he carried traces of these products into his home and his wife washed his work clothes separately.

The mother took acetaminophen in the periconceptional period and occasionally during the first trimester for toothaches. She began taking iron in the 12^th^ gestational week. No use of other medications, alcohol, tobacco, vitamins or pharmaceutical products was reported. Dietary intake of folates during the periconceptional period was estimated to be 500ug/day. On 30 September 2006, midway through the 5^th^ gestational week, the mother received a dental X-ray to diagnose caries, without radiation protection.

## Discussion

The cloacal bladder exstrophy-epispadias complex (CBEEC) represents a collection of congenital malformations caused by failed mesenchymal development during the first trimester of gestation. CBEEC is a continuum, representing different levels of severity within the same spectrum [10]. Before the 5^th^ week of embryonic development, the urinary and gastrointestinal tracts and genitals drain into a common chamber. At the 7^th^ week, the cloaca is divided into an anterior chamber, the primitive urogenital sinus, and a posterior chamber, the rectum. Transient communication exists between the two parts of the cloaca below the urorectal septum. By the end of the 8^th^ week, division normally occurs.

Different malformations of the primitive urogenital sinus in humans, such as cryptorchidism, hypospadias and epispadias, have been associated with exposure to hormonal pesticide disruptors during critical developmental periods [7,8].

The mother of our patient may have been exposed to various pesticides, but it is those which she sprayed in her home that reached toxic levels. Pyrethroids have a more irritant effect on the respiratory mucosa and conjunctiva than pyrifoxen and explain the mother's symptoms [11]. The qualitative method which we used was capable of detecting pyrethroids and pyriproxyfen in the child's urine.

Pyriproxyfen is a hormonal insecticide analogue to high estrogenic activity designed to interfere with the insects' developmental processes. When treated with pyriproxyfen, both female and male insects yield young with physical abnormalities [12,13]. However, animal experiments do not show prenatal developmental toxicity in the presence of maternal toxicity [14]. Tetramethrin and cyphenothrin are synthetic pyrethroid insecticides. There is evidence that pyrethrins are associated with endocrine disruption. Direct measurements of serum thyroid hormones as well as histopathologic alterations observed in the thyroid gland itself give rise to concern for potential endocrine disruption of pyrethrins. Recent studies suggest that permethrin may have estrogen-like effects on female rats and anti-androgen-like effects on males which may be correlated with sertoli cell and spermatogenic epithelium impairment [15].

There are confounding factors to take into account. We believe the contribution of ionizing radiation is less relevant than pesticide use because of the small estimated dose absorbed by the fetus and the timing of the exposure (in the 5th embryonic week; we would expect a more severe malformation such as cloacal exstrophy). Our patient was also exposed to pesticides as a fetus through the mother's diet, although the magnitude was smaller than the residential pesticide exposure. In some cases, pesticides used for vector control may be present in, or deliberately added to, drinking water. Data from health authorities shows that drinking water at our patient's home was free of pesticides.

We believe that the PEH is the best clinical tool to approximate the etiology of rare pediatric diseases. However, it is not sufficient to meet the Bradford-Hill causal criteria. The individual risk assessment for these patients is a complex process that requires specific diagnostic abilities. Although the mother was clinically poisoned during organogenesis, and the potential relationship between pesticide exposure and BE is plausible, we must still be cautious.

Our work also serves to highlight several limitations and challenges of the assessment of exposure to hormonal pesticide disruptors that need to be addressed. We must complete exposure classification using direct measures, improve interpretation of complex dose-response relationships, estimate exposure by taking into account the highly heterogeneous chemical classes implicated, and develop biomarkers that allow investigators to quantify exposure to mixtures of endocrine disruptors to be able to differentiate between their effects.

It is difficult to attribute a cause to a condition when so many factors remain unknown. Despite the apparent lack of conclusive evidence, we should err on the side of caution and apply our scientific knowledge to protect current and future generations. It is important to give specific pest control recommendations to eliminate or diminish the pesticides used at home. In addition, we must remember to look for alternatives to ionizing radiation during pregnancy and only conduct radiologic examinations during the first postmenstrual week in any woman of childbearing age. The PEH not only helps register the RFs involved but also to improve the home environment and quality of life.

## Conclusions

A detailed and carefully conducted PEH that includes information regarding home pesticide use should be carried out for all children with BE. The PEH is a useful tool that can help improve the home environment quality of life and allows clinicians to give specific recommendations for healthier pregnancies.

## Abbreviations

BE: bladder exstrophy; CBEEC: cloacal bladder exstrophy-epispadias complex; PEH: pediatric environmental history; RFs: risk factors.

## Consent

Written informed consent was obtained from the patient's parents for publication of this case report and any accompanying images. A copy of the written consent is available for review by the Editor-in-Chief of this journal.

## Authors' contributions

JAOG, MM and KR conducted the PEH and analyzed and interpreted the patient data regarding the disease. GCZ conducted the morphological study. All authors read and approved the final manuscript.

## Competing interests

The authors declare that they have no competing interests.
